# Long-Term Protection and Serologic Response of European Sea Bass Vaccinated with a Betanodavirus Virus-Like Particle Produced in *Pichia pastoris*

**DOI:** 10.3390/vaccines9050447

**Published:** 2021-05-02

**Authors:** Sofie Barsøe, Anna Toffan, Francesco Pascoli, Ansgar Stratmann, Tobia Pretto, Andrea Marsella, Mériem Er-Rafik, Niccolò Vendramin, Niels J. Olesen, Dagoberto Sepúlveda, Niels Lorenzen

**Affiliations:** 1 National Institute of Aquatic Resources (DTU AQUA), Technical University of Denmark, 2800 Lyngby, Denmark; sofhan@aqua.dtu.dk (S.B.); niven@aqua.dtu.dk (N.V.); njol@aqua.dtu.dk (N.J.O.); dsep@aqua.dtu.dk (D.S.); 2 Istituto Zooprofilattico Sperimentale delle Venezie (IZSVe), 35020 Legnaro, Padua, Italy; atoffan@izsvenezie.it (A.T.); fpascoli@izsvenezie.it (F.P.); tpretto@izsvenezie.it (T.P.); AMarsella@izsvenezie.it (A.M.); 3 W42 Biotechnology GmbH, 44227 Dortmund, Germany; ansgar@marinnovac.com; 4 National Center for Nano Fabrication and Characterization (DTU Nanolab), Technical University of Denmark, 2800 Lyngby, Denmark; merafik@dtu.dk

**Keywords:** VLP vaccine, recombinant vaccine, betanodavirus, efficacy, correlate of protection

## Abstract

Viral Nervous Necrosis (VNN) causes high mortality and reduced growth in farmed European sea bass (*Dicentrarchus labrax*) in the Mediterranean. In the current studies, we tested a novel *Pichia*-produced virus-like particle (VLP) vaccine against VNN in European sea bass, caused by the betanodavirus “Red-Spotted Grouper Nervous Necrosis Virus” (RGNNV). European sea bass were immunized with a VLP-based vaccine formulated with different concentrations of antigen and with or without adjuvant. Antibody response was evaluated by ELISA and serum neutralization. The efficacy of these VLP-vaccine formulations was evaluated by an intramuscular challenge with RGNNV at different time points (1, 2 and 10 months post-vaccination) and both dead and surviving fish were sampled to evaluate the level of viable virus in the brain. The VLP-based vaccines induced an effective protective immunity against experimental infection at 2 months post-vaccination, and even to some degree at 10 months post-vaccination. Furthermore, the vaccine formulations triggered a dose-dependent response in neutralizing antibodies. Serologic response and clinical efficacy, measured as relative percent survival (RPS), seem to be correlated with the administered dose, although for the individual fish, a high titer of neutralizing antibodies prior to challenge was not always enough to protect against disease. The efficacy of the VLP vaccine could not be improved by formulation with a water-in-oil (W/O) adjuvant. The developed RGNNV-VLPs show a promising effect as a vaccine candidate, even without adjuvant, to protect sea bass against disease caused by RGNNV. However, detection of virus in vaccinated survivors means that it cannot be ruled out that survivors can transmit the virus.

## 1. Introduction

Viral nervous necrosis (VNN), or viral encephalo- and retinopathy (VER), is a viral disease of many marine and freshwater fish, including European sea bass (*Dicentrarchus labrax*), causing an infection of the central nervous system with spiraling swimming pattern, loss of buoyancy control and darkening of the skin being some of the most evident clinical manifestations [1,2,3,4]. The etiological agent is *Betanodavirus*, a small (25–30 nm) non-enveloped icosahedral virus belonging to the family Nodaviridae. The genome is bi-segmented, with two single-stranded, positive-sense RNA molecules: The RNA 1 and the RNA2. RNA1 (3.1 kb) encodes the RNA-dependent RNA polymerase (app. 100 kDa) involved in the viral replication and with a + 1 shift in reading frame also a subgenomic RNA3 (0.4 kb) is translated, encoding for protein B2 which suppress cellular RNA interference [5]. RNA 2 (1.4 kb) encodes the capsid protein (app. 42 kDa), comprised of an N-terminal arm, a shell (s)-domain and a protrusion (p)-domain [6]. The p-domain determines the host specificity of the virus and harbors the variable T4 region used to classify the *Betanodavirus* into four species: red-spotted grouper (RG) nervous necrosis virus (NNV), striped jack-(SJ)NNV, tiger puffer-(TP)NNV and barfin flounder (BF)NNV. The species can re-assort the two RNA segments, giving rise to re-assortant strains with, e.g., RNA1 from RGNNV and RNA2 from SJNNV (RG/SJNNV) [7]. The four species are antigenically different and have been divided into three serotypes; RGNNV belong to serotype C, while SJNNV and TPNNV are serotypes A and B, respectively. BFNNV has been classified both as serotype C [8,9] and B [10].

The *Betanodavirus* is very resistant in the aquatic environment, where it can survive on boats, equipment, etc., posing a threat to both the farmed fish and wild fish, since the virus has more than 120 host species [9,11]. Meanwhile, the presence of virus in the wild fish populations acts as a reservoir with continuing introduction to the farms. Different vaccine prototypes have been developed such as inactivated, recombinant, and DNA vaccines, and in general it is possible to induce protective immunity against disease [12,13,14,15]. In 2018, an inactivated oil-adjuvanted vaccine was registered for use in European sea bass (“ALPHA JECT micro^®^ 1 Noda”, Pharmaq [16]), and shortly after another followed (“ICTHIOVAC^®^ VNN”, Hipra [17]). These are now available in selected Mediterranean countries for prophylactic treatment of European sea bass against VNN. However, beside the information provided by the producers, no documentation of the potency of these vaccines is available, and previous studies with intra-peritoneal administration of mineral oil adjuvanted vaccines to European sea bass have shown that the oil can cause moderate lesions in the abdominal cavity [18]. In addition, studies in other fish species have related the presence of abdominal lesions to reduced growth and welfare [19,20]. Therefore, we still believe, that there is a need for new innovative vaccines against VNN.

The simple structure of betanodavirus capsid protein formed by only one protein encoded by the RNA2 makes it ideal for the production of virus-like particles (VLPs) in expression systems such as yeast, bacteria, mammalian, insects or plants, which has also been achieved with success [6,21,22,23,24]. Being the same size, and with similar immunogenicity and antigenicity as the native virion, VLPs are recognized and reacted against by the immune system [25]. Furthermore, the repetitive sequence of antigens on the surface not only enhance the activation of the innate immune system, but often also induce a strong antibody response [25]. VLPs are, therefore, in general thought of as promising vaccine candidates [25,26,27,28]. Thiéry et al. (2016) [29] showed promising protection of a baculo-virus expressed RGNNV VLP in a vaccination-challenge experiment with European Sea bass approximately 1 month after vaccination. Likewise, a recombinant vaccine with *E. coli* expressing RGNNV capsid showed protection in intramuscular (IM) challenge 30 days post-vaccination [13]. We here present an RGNNV VLP produced in an eukaryotic yeast expression system (*Pichia pastoris)*, which is cost-efficient, easy to upscale and with low risk of contamination with endotoxins or GMO-vectors, as is possible when *E. coli* or baculo-virus expression systems are used, respectively [30]. Correct size, structure and integrity of VLPs were confirmed by transmission electron microscopy and Western blot. The immunogenic properties of the VLP were tested through intraperitoneal immunization of European sea bass in two studies. Study 1 aimed at investigating the dose-response of the VLP-vaccine, both by evaluation of specific and neutralizing antibodies in serum 27 and 57 days post-vaccination and in an experimental IM challenge 2 months post-vaccination (p.v.). The second study (Study 2) included a time-course experiment where the protection induced by the VLP vaccine antigen with or without a water-in-oil adjuvant was evaluated by IM challenge with RGNNV 1 and 10 months p.v., respectively.

## 2. Materials and Methods

### 2.1. VLP Production and Purification 

*Pichia pastoris* (*Komagataella phaffii*) strain W42-A and plasmid pWM15 were provided by W42 GmbH (Dortmund, Germany). The expression cassette in the plasmid comprises a strong yeast promoter, P*w42-2,* in combination with a yeast-specific transcription termination region (aox1TT). The *ble* gene from *Streptomyces* sp., providing resistance against the antibiotic Zeocin (Invivogen) in *E. coli* and *P. pastoris*, was used as a selection marker.

For the production of the RGNNV capsid protein, the coding gene sequence was synthesized as DNA with optimized codon usage for *P. pastoris* and inserted downstream the P*w42-2* promoter in pWM15 using restriction sites for the enzymes *Eco*RI and *Xba*I at the 5′- and 3′-end, respectively. Since the open reading frame did not encode any secretion signals (as for the parental RGNNV capsid protein gene), expressed capsid protein would be expected to accumulate in the cytoplasm of expressing cells. The construction of the capsid*-*expressing plasmid was carried out in *E. coli*. The resulting expression plasmid pWVE1-2 was transferred to *P. pastoris* in linearized form by standard electroporation to enable the genomic integration of the plasmid in the P*w42-2* gene locus of the yeast. Capsid-producing clones were achieved by selection on agar plates with increasing Zeocin concentration. Correct genomic integration of the capsid expression cassette was confirmed by PCR. Using synthetic medium and induction by mixed feed consisting of glycerol/methanol, the best producer clone was identified after shake flask cultivation by standard Western blot procedure. To produce the required amount of recombinant RGNNV VLP, a lab-scale fermentation in a 4 l working volume was carried out in defined synthetic medium controlled by optimized mixed feed of glycerol/methanol. *P. pastoris* cells from fermentation were harvested by centrifugation and freeze dried after harvest and kept at 4 °C until further use.

Preparation of VLP was carried out using a standardized procedure. Freeze-dried cells were dissolved in buffer and broken mechanically by glass beads using the Bead Beater (Bio Spec Products Inc., Bartlesville, OK, USA) according to the manufacturer’s specifications. Cell debris was removed by centrifugation (10,000× *g*, 15 °C, 30 min). The soluble fraction was used for the enrichment of VLP by specific precipitation followed by sterile filtration (0.2 µm). Final protein concentration was determined by Bradford assay.

### 2.2. Transmission Electron Microscopy and Cryo-Transmission Electron Microscopy

The VLPs were observed in native state by cryo-transmission electron microscopy (cryo-TEM). To make the observations, a volume of 3 μL of VLP solution was placed onto glow-discharged (Q150T, Quorum, East Sussex, UK) grids (Lacey Formvar/Cu grids, SPI Supplies, West Chester, PA, USA) and plunge frozen into liquid ethane, cooled with liquid nitrogen, using a GP2 (Leica, Wetzlar, Germany), with the following settings: 100% humidity, 277 K and blot time 3 sec. Imaging was done using a Tecnai G2 (FEI, Hillsboro, OR, USA) microscope at 200 kV. Images were recorded with a camera TemCam-XF416 (TVIPS, Gauting, Germany).

### 2.3. Western Blot

An SDS-page gel (RunBlue SDS gel 4–12%, Abcam plc, Cambridge, UK) was run with non-reducing loading buffer (180 V, max 0.18 A, 1.5 h) with VLP (100 µg/mL), ultracentrifuged live RGNNV (strain 283.2009, MilliQ water and a marker (precision protein standards, 113–21.5 kDa, Broad Range, 6 bands, BIO-RAD, Hercules, CA, USA)). The ultracentrifuged virus was prepared from a batch of live virus/L15-media solution grown on SNN-1 cells [31] with a starting titer on 5.9 × 10^8^ TCID_50_ (determined by Reed-Muench [32]). The solution was ultracentrifuged (25.000 RPM, 4 °C, 18 h, Rotorhead SW28, Optima XE-90 Ultracentrifuge, Beckman Coulter, Brea, CA, USA), the pellet and a few ml of supernatant were retrieved and re-suspended in IDTE buffer, where after the solution was ultracentrifuged again (45.000 RPM, 4 °C, 3 h, Rotorhead SW55 TI, Optima XE-90 Ultracentrifuge, Beckman Coulter, Brea, CA, USA). The final pellet was re-suspended in 1 ml IDTE buffer and stored at −80 °C until use.

The protein bands were transferred by a semi-dry transfer (Trans-Blot SD Semi-Dry Transfer Cell (BIO-RAD, Hercules, CA, USA), 18 V, 0.4 A, 1 h) to a PVDF membrane. The membrane was blocked with PBS-Tween20 + 5% skimmed milk and immuno-stained with rabbit-anti-noda (1:500 in PBS + 1%BSA) (Antiver, IZSVe, Padua, Italy [10]) followed by swine anti-rabbit conjugated with HRP (1:1000 in PBS + 1%BSA) (p217, Dako, Glostrup, Denmark). The binding of antibodies to antigens was evaluated by the detection of brown precipitates after development with DAB substrate for 5–10 min.

### 2.4. Fish Experiments

Two experimental sites were used, the first being Istituto Zooprofilattico Sperimentale delle Venezie (IZSVe), Padua, Italy (Study 1), and the second being The Unit for fish and shellfish diseases, the National Institute of Aquatic Resources, Technical University of Copenhagen (DTU AQUA), Denmark (Study 2). Study 1 was a dose-titration study investigating the kinetics of serology with ELISA and SN and survival after experimental challenge 2 months post-vaccination. Study 2 tested if formulating with an adjuvant enhanced or prolonged the induced protection against VNN in an experimental challenge at 1 and 10 months post-vaccination. During the 10 months immunization period in Study 2 (Group 1) the fish were kept on a remote site at “Den Blå Planet”, the National Aquarium Denmark (Copenhagen, Denmark). An overview of the two studies and the different analysis performed, described in detail in the following sections, is summarized in Table 1.

### 2.5. Vaccine Preparations

The VLP was administered as aqueous solution and diluted in PBS to the desired concentrations. The stock VLP had a protein concentration of 400 µg/mL (Bradford assay). The formulations used in Study 1 were 1.25, 5 and 20 µg VLP/fish (average 34 fish/group) and in Study 2: 6 µg (±adjuvant) and 20 µg (average 55 fish/group). The adjuvanted formulation was made by diluting the stock VLP in Montanide ISA 761 (Seppic, Cedex, France) (3:7) and homogenizing by ultra-sonication using Soniprep 150 Plus (MSE, London, UK) at maximum amplitude for 2 × 30 s on ice. In both studies, a group receiving PBS (±adjuvant in Study 2) and another receiving a commercial vaccine (inactivated NNV with mineral oil adjuvant) served as negative and positive control groups, respectively. All vaccines were administered intraperitoneal (IP) at a volume of 50 µL. In addition, a tank of non-challenged fish was kept in both studies.

### 2.6. Fish and Setup

Study 1: European sea bass (weight: 29 ± 2 g) from an uncharacterized population were obtained from a commercial farm and quarantined into the IZSVe facilities until initiating the experiment. During the experiment (≈3 months) the fish were housed in separated saltwater tanks (300 L, 25‰) equipped with heater, aerator and recirculation pumps. Water temperature was set at 20 °C during vaccination and immunization period (2 months, 1180 degree days (dd)) and increased to 25 °C after challenge. The fish were fed daily with a commercial diet (2% Body Weight). The sea bass were anesthetized in MS-222 (Tricaine Pharmaq, Overhalla, Norway) and vaccinated as described above.

Study 2: European sea bass from an Atlantic population [33,34] was retrieved from MARBEC, Université Montpellier, CNRS, Ifremer, IRD, Palavas-les-Flots, France (weight: 5 g ± 2 g). Upon arrival, the fish were divided in two groups (“Group 1” and “Group 2”). A week after arrival, the fish in Group 1 were anesthetized in 0.04% benzocaine solution and received one of the six treatments (as described above) and were left for 10 months in one tank pr. group (600 L, artificial saltwater (30‰), 12 °C) (immunization period: 10 months/3600 dd). Group 2 was kept untreated for 9 months in one tank (180 L, artificial saltwater (15‰), 19 °C) and then vaccinated with the same reagents as the first group (Immunization period: 1 month/570 dd). Upon vaccination, the fish in Group 2 were tagged according to treatment by subcutaneous injection of fluorescent elastomers (VIE tag—Northwest Marine Technology) caudal to the eye (left or right side, three colors) and kept in the same tank during immunization.

**Figure 1:**
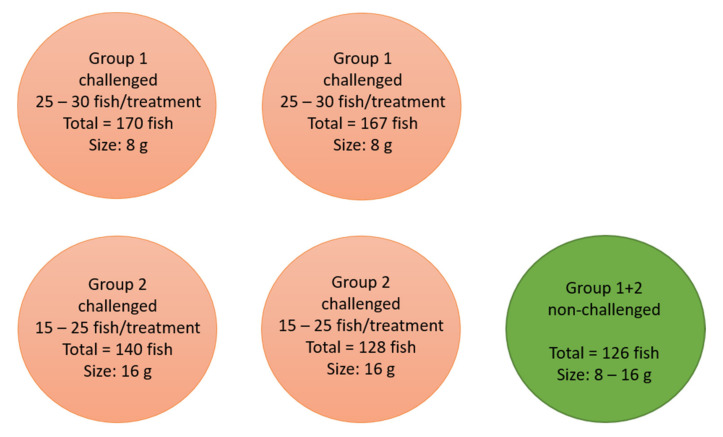
Setup during challenge in Study 2 (DTU). The fish were kept in cylindrical 180 L tanks with aerated saltwater (12‰) at 26 °C (±1 °C). A bio-filter pump was installed in each tank and the wastewater was UV and heat treated.

The week before the challenge, all fish in Experiment 2 were moved to the infection unit at DTU and kept in 180 L tanks with artificial saltwater (12‰) at 19 °C while gradually increasing the temperature to 26 °C up to the day of challenge (setup explained in Figure 1). As a result of the different rearing conditions, the previous 10 months, the mean weight of Group 1 and 2 at this time was approximately 8 and 16 g, respectively.

### 2.7. Sampling

In Study 1, at Day 27 post-vaccination (p.v.), 5 fish pr. group were non-lethally blood sampled and marked individually with fluorescent elastomer tagging after blood sampling (Fish 1–5) (VIE tag—Northwest Marine Technology, Anacortes, WA, USA) (Figure S1). At Day 57 p.v., 10 fish were blood sampled non-lethally; Fish 1–5 from Day 27 and 5 new fish that were also tagged individually (Fish 6–10).

In Study 2, 10 (Group 1) or 5 (Group 2) fish pr. treatment were euthanized and blood sampled just before the challenge, corresponding to 10 or 1 months post-vaccination.

### 2.8. Virus and Challenge

RGNNV (strain 283.2009 [35]) was used for challenge of vaccinated fish in both studies, though in Study 1 the virus was cultured on E-11 cells [36], a clone of SNN-1 cells [31], and in Study 2 the virus was grown on SNN-1 cells. The virus was harvested when cytopathic effect (CPE) was complete and the suspension with cell debris was frozen at −80 °C overnight, thawed and centrifuged to remove cell debris, followed by storage at −80 °C until challenge. At the day of challenge, the virus batch was thawed and mixed to ensure homogeneity, and diluted in cell culture medium to obtain the desired titer. After the challenge, an aliquot of virus suspension was titrated on a 96-well plate (Falcon Primary, Thermo Fisher Scientific, Waltham, MA, USA) with a 1-day-old cell-monolayer to confirm the inoculated titer. The plate was incubated for 10 days and the final titer was determined using TCID_50_ “Reed-Muench” method [32].

In Study 1, the challenge was performed 2 months post-vaccination. The fish were anesthetized in MS-222 and injected IM with 2 × 10^5^ TCID_50_/fish (100 µL, 27 G needle). In Study 2, the challenge was performed 1 and 10 months post-vaccination, respectively. The fish were anesthetized in benzocaine solution (0.04%) and injected IM with 1 × 10^5^ TCID_50_/fish (50 µL, 27 G needle).

In both studies, the fish were checked at least daily and moribund fish were euthanized. When the tagged fish in Study 1 were euthanized or succumbed to the infection they were sampled for titration of virus in their brain on cell culture (fish 6–10). The experiment was terminated after approximately 4 weeks and surviving fish were euthanized and counted as survivors. A selected number of survivors in Study 1 (up to 10, unless fewer survived) were sampled by drawing blood and collecting brain in sterile media. If the tagged fish survived, these were prioritized in the sampling. In Study 2, the brain was sampled from 6 dead/euthanized fish (3 per tank) per treatment/group and from 6 survivors (3 per tank) per treatment/group for detection of virus with RTqPCR. The samples were stored in RNA later at −80 °C until further analysis.

### 2.9. Titration of Virus from the Brain (Study 1)

A total of 250 mg of brain was collected in an Eppendorf tube and homogenized using small sand grains and 500 µL sterile MEM media. Following centrifugation, 150 µL of the supernatant was treated with a mixed commercial Pen/Strep antibiotic solution (Merck, Kenilworth, NJ, USA) (1:10) (20 h, 4 °C) and the following day a 10-fold dilution, starting at concentration 1:10 was inoculated on a monolayer of E-11 cells in duplicates on a 96-well tray and incubated at 25 °C. The final CPE was read on Day 10 and the viral titer was calculated as TCID_50_/g.

### 2.10. RT-qPCR

In Study 1, 250 µL of homogenized brain supernatant (from “Titration of virus from the brain”) was transferred to 300 µL lysis buffer and analyzed with qualitative RTqPCR against nodavirus RNA 1, as previously described [37]. In Study 2, a multiplex RTqPCR was used to detect both RGNNV RNA1 (primers as in [37]), and the housekeeping gene elongation factor 1 alpha (primers as in [38]). The Cq value of both targets was thereafter used to make a relative quantification of the amount of RNA1 in each sample, as described by Barsøe et al., 2021 [34].

### 2.11. Immunohistochemistry (Study 1)

The brain was carefully dissected from four moribund fish from the group inoculated with PBS (negative control) and five survivor fish in the VLP high-dose group and fixated in buffered formalin for immunohistochemistry (IHC), as previously described [39].

### 2.12. ELISA

The collected sera were tested for specific antibodies against RGNNV in ELISA [40]. Briefly, the plates were coated with ultracentrifuged formalin inactivated RGNNV (strain 283.2009) diluted 1:100 in carbonate-bicarbonated pH 8.9 buffer (incubated 20 h, 4 °C) and thereafter blocked with PBS + 3% bovine serum albumin (BSA). The plasma/sera were analyzed in 1:100 dilution in duplicates with rabbit anti-sea bass IgM (1:6000) (IZSVe, Padua, Italy) and goat anti-rabbit-HRP (1:3000) (Sigma-Aldrich, St. Louis, MO, USA) as secondary and tertiary antibodies, respectively. TMB was added to activate the HRP and the reaction was stopped with H_2_SO_4_ after 5 min when a clear color change was evident. The absorbance was measured at 450 nm in duplicate wells and the average was calculated. A positive and four negative control samples was included on each plate and these were used to calculate the sample/positive ratio (S/P):

$$S/P=\frac{mean\;ABS\;\left(sample\right)-mean\;ABS\;\left(neg\;control\right)}{mean\;ABS\;\left(pos\;control\right)-mean\;ABS\;\left(neg\;control\right)}\times 100$$

An *S/P* < 42% was considered negative, from 42–56% was considered doubtful and >56% was considered positive.

### 2.13. Serum Neutralization (Study 1)

Sera were heat treated at 45 °C for 30 min to inactivate complement and thereafter a two-fold dilution series was made, starting from 1:10. Infective RGNNV 283.2009 (100 TCID_50_/well) was added at equal volume to each of the dilutions and incubated overnight at 4 °C. The day after, the serum/virus samples were transferred to a 96-well plate with 1-day-old E-11 cells monolayer and incubated at 25 °C for 10 days, where wells with CPE were noted. All samples were run in duplicate, and the final titer was the dilution at which both duplicates were negative for CPE. When no inhibition of CPE was seen in any of the dilutions, the titer was denoted as <1:20. In each setup, a control plate with titration of virus and positive reference rabbit-anti-noda serum (Antiver, IZSVe, Padua, Italy [10]) was included to secure reproducibility.

### 2.14. Statistics

Statistical analysis was performed in STATISTICA 13 (TIBCO Software) and R (ver. 4.0.3) [41]. Kaplan–Meier survival curves were conducted with the packages “survival” (ver. 3.2-7) [42,43] and “survminer” (ver. 0.4.8) [44] in R for each of the experiments. In Study 1, Pearson’s Chi-squared test (“chisq.test“) was done to evaluate if the likelihood of the observed different survival% in the groups arose by chance or was a result of the treatments. Post-hoc comparisons were done with Bonferroni correction for multiple comparisons.

Study 2 was first evaluated with a logistic regression (generalized linear model/”glm()”) to determine if the factors in Study 2 (adjuvant and tank) influenced the probability of surviving (p(surv)) until the end of the study;

$$logit\left(p_{surv}\right)=\alpha +\beta_{Treatment}+\beta_{adjuvant}+\beta_{tank}$$

Stepwise model reduction was performed until the most simple model was achieved with only significant factors. Odds ratio (OR) of survival was calculated from the β extracted from the model; OR = exp(β). To validate the model, we used the function to predict the p(surv) at the two time points and plotted the predicted values in a graph with the actual p(surv) data from the two replicate tanks. If predicted and actual values fitted, we assumed that the model was valid.

Secondary, Pearson’s chi-squared test followed by pairwise comparison was used to compare the treatment groups in Study 2, Group 2, as done in Study 1.

The relative amount of RNA1 in the brain of survivors from each treatment in Study 2 was compared with an ANOVA (linear model/”lm()”) in R.

Figures were made with ggplot2 [45], unless otherwise stated. *p*-values ≤ 0.05 were considered significant.

## 3. Results

### 3.1. Production and Charachterization of the VLP

**Figure 2:**
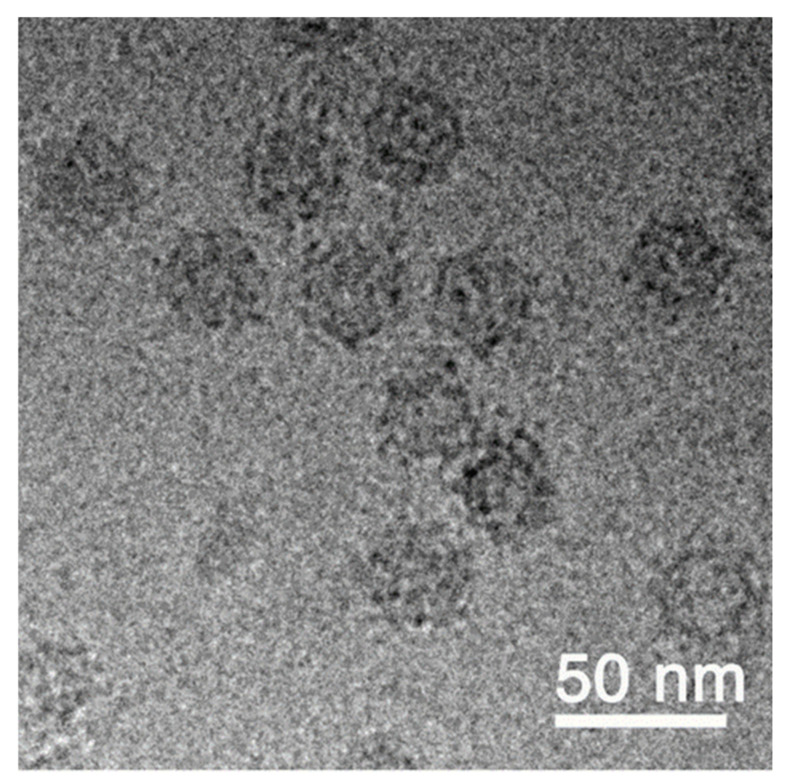
Visualization of RGNNV VLPs by cryo-TEM.

Successful production and enrichment of VLP were characterized by cryo-transmission electron microscopy (Figure 2). The VLP solution was adjusted with buffer to 400 µg/mL as stock for the described assays and challenge experiment. The native structure of VLP observed by cryo-TEM showed a spherical shape (~30 nm in diameter) decorated with several high-density nanoclusters. Preliminary results of the three-dimensional reconstruction from cryo-TEM images with 12 Ångstrøm resolution shows an icosahedral (T = 3) structure with high-density nanoclusters corresponding to the P-domain, as described by Chen et al. (2015) [6].

**Figure 3:**
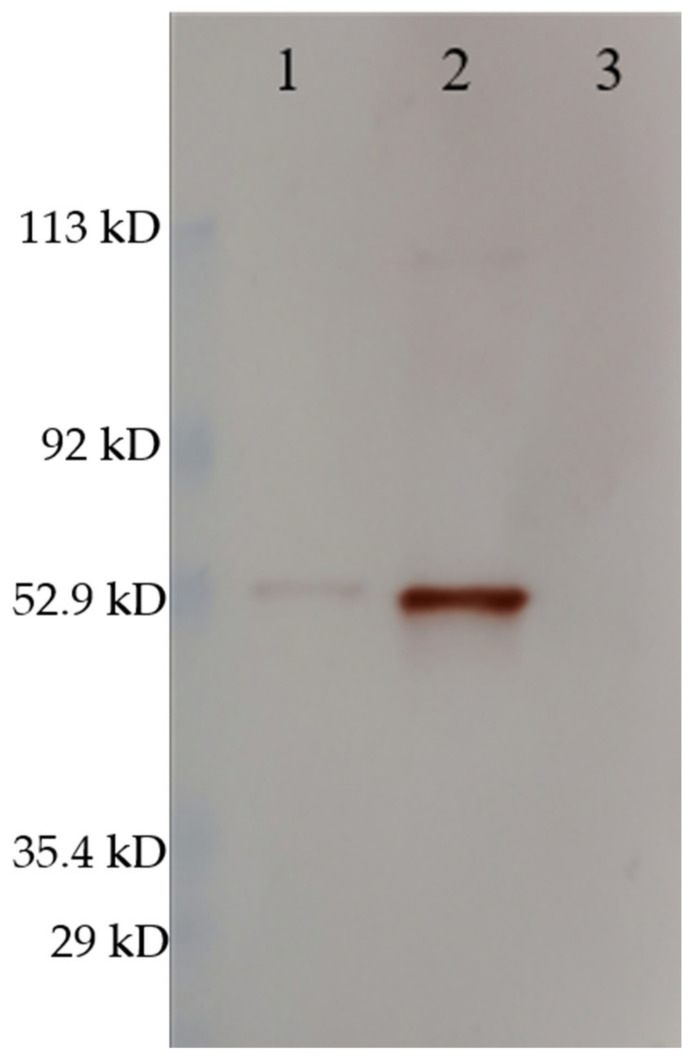
Western blot stained with rabbit-anti-nodavirus. (1) RGNNV (strain 283.2009), (2) RGNNV VLP, (3) MilliQ H_2_O. The RGNNV sample was concentrated virus obtained by ultracentrifugation of cell culture supernatant and was not quantified since impurities from the cell culture medium would contribute to total protein. The molecular weight indicated by the marker must be interpreted with caution since the marker was pre-stained (as described in Section 2.3). Uncropped photos are shown in the supplementary material (Figure S2).

The Western blot confirmed the same size of the VLPs and RGNNV virus (strain 283.2009) capsid proteins (Figure 3) as well as antigenic similarity in terms of the polyclonal rabbit-anti-nodavirus antibody binding to both. The difference in the intensity of the bands reflects different concentrations of VLP and virus, as this was not normalized.

### 3.2. ELISA

**Figure 4:**
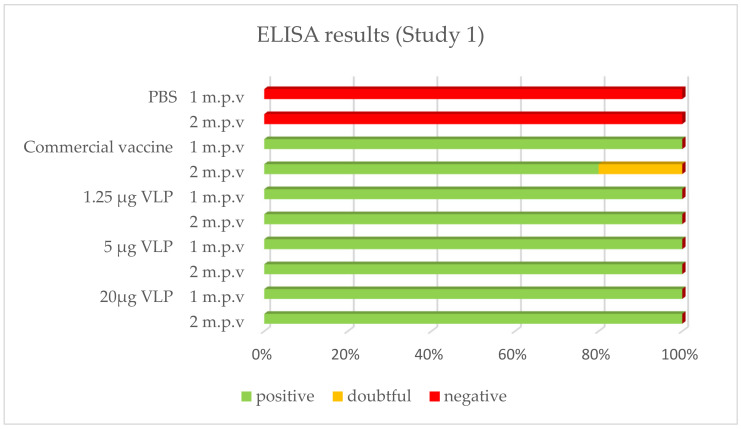
Prevalence of -specific IgM in ELISA (Study 1). Serum from vaccinated fish was sampled 27 days (“1 m.p.v.”, *n* = 5) and 57 days (“2 m.p.v.”, *n* = 10) after vaccination.

**Figure 5:**
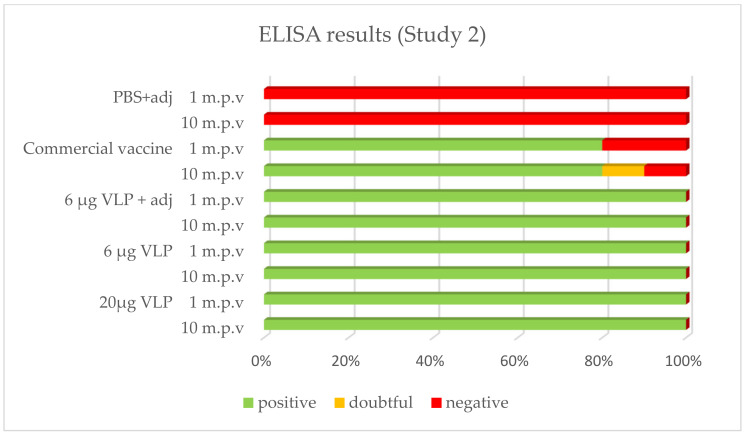
Prevalence of RGNNV-specific IgM in ELISA (Study 2). Plasma from sea bass 1 (*n* = 3–5) or 10 (*n* = 10) months post-vaccination (m.p.v).

After vaccination, 100% of the sea bass immunized with the VLP formulations tested positive by ELISA whatever the time of sampling (1, 2 or 10 month p.v.). With the commercial vaccine, 100% of fish tested positive after 1 month in Study 1, while this proportion was 80% in Study 2. In both studies, 80% was positive in the latter sampling 2 or 10 months p.v., respectively. The positive, doubtful or negative detection (based on criteria set up in the methods) of specific antibodies against RGNNV in ELISA is presented in Figure 4 and Figure 5.

### 3.3. Serum Neutralization (Study 1)

**Figure 6:**
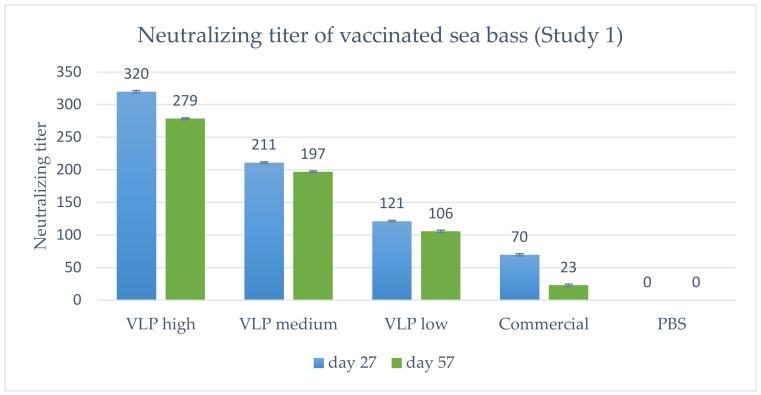
Geometric mean neutralizing titer in vaccinated fish on Day 27 (*n* = 5) or 57 (*n* = 10) post-vaccination, Study 1. VLP high = 20 µg/fish, medium = 5 µg/fish or low = 1.25 µg/fish. Bars indicate geometric standard deviation. The lowest serum dilution analyzed was 1:20, which is why a negative sample was <1:20. When calculating the geometric mean titer, these were set to a value of 10, except in the PBS group where all were <1:20, and set as 0.

The titer of neutralizing antibodies was determined and the geometric mean titer calculated (Figure 6). In general, a dose-dependent response in titer was detected, peaking at 1 month p.v. (Day 27) and declining slightly at 2 months p.v. (Day 57). Notably, all VLP concentrations (20, 5 and 1.25 µg) induce higher neutralizing antibodies than the commercial vaccine.

### 3.4. Survival

**Figure 7:**
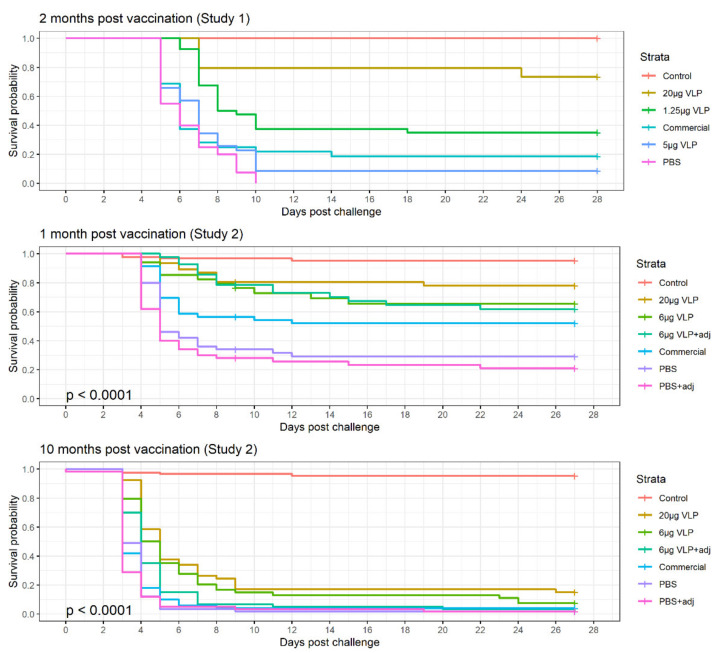
Kaplan–Mayer survival curve of the three experimental challenges with intramuscular injection of RGNNV.

In the experimental challenges, clinical signs of disease were evident from Day 4 post-challenge, with a peak in incidence during Day 5–10 and stabilizing on Day 15 p.c (Figure 7). In Study 2, on Day 9 of the study, 21 fish were lost in one of the tanks with Group 1 due to equipment failure. These were a mix of all treatment groups and counted as survivors until Day 9, when they were censored for the analysis. In the logistic regression where the end survival probability is compared (Day 27 p.c.), these were removed from the dataset.

### 3.5. Statistical Evaluation

In Study 1, survival in the group receiving VLP 20 µg was significantly different from the other groups (*p* > 0.001), while the VLP 5 µg, 1.25 µg and commercial vaccine were not significantly different from each other.

The logistic regression of the results in Study 2 revealed that the tank variability was insignificant (*p* = 0.52), allowing us to combine the data for the replicate tanks in the analysis. Adjuvant was also insignificant (*p* = 0.26) and thus removed from the model, combining the adjuvanted and non-adjuvanted treatments (“VLP 6 µg ± adj” = “VLP 6 µg”, and “PBS ± adj = PBS”). There was no interaction between Time and Treatment. Thus, the two final significant factors for the probability of survival ((*p*(surv)) was the time post-vaccination and the treatment (*p* < 0.001). All three treatments (VLP 20 µg, VLP 6 µg and commercial vaccine) significantly increased the odds of surviving compared to the group receiving PBS (Table 2). Even though a higher chance of survival was seen in the VLP high-dose group, this is not significantly different from the two other treatments in this model as the 95% CI overlap (Table 2). At 10 months after vaccination (3600 dd), the chance of surviving was significantly reduced in all groups (OR = 0.7 [0.7–0.8]).

The model was validated by calculating the predicted p(surv) with the model and plot it with the observed p(surv) in the two replicate tanks, which was within the predicted intervals (Figure S3). 

Comparison of the treatments in Study 2, Group 2 (challenged 1 month post-vaccination) with Pearson’s chi-squared test showed a significant difference in survival between the groups VLP 20 µg, 6 µg and commercial vaccine and the group receiving PBS (*p* > 0.001, *p* = 0.031, *p* = 0.021, respectively), while VLP 20 µg was also significantly different from the commercial vaccine (*p* = 0.034). The comparison was only done for the non-adjuvanted groups.

### 3.6. Detection of Virus in Brain-Tissue

**Figure 8:**
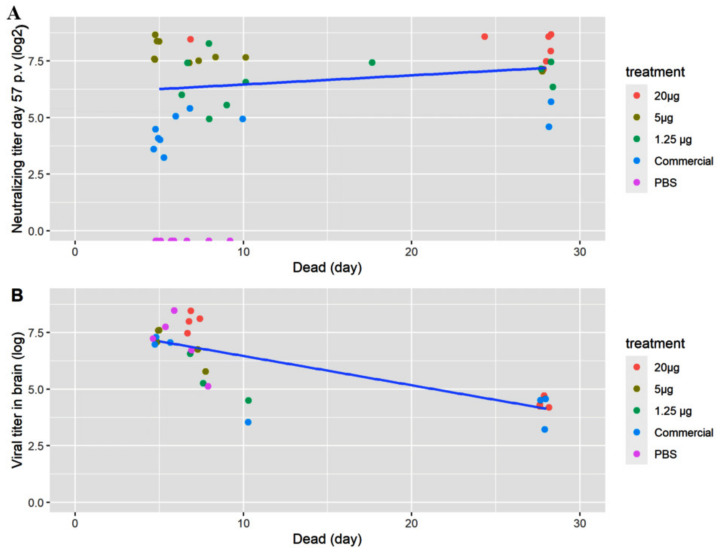
Data on individual fish. (**A**) Neutralizing titer before challenge and (**B**) RGNNV virus titer (TCID50/g) isolated from brain tissue from dead/moribund fish as a function of the day the fish was terminated/diseased. The titers were log transformed to obtain linearity. The colors indicate the treatment (20, 5 or 1.25 µg VLP, commercial vaccine or PBS).

In Study 1, the tagged fish were prioritized in the sampling of brain for virus isolation. No tagged fish from the group receiving 20 µg VLP was diseased, which is why four of the non-tagged diseased fish from Day 7 were titrated instead. When sampling the survivors, only the groups “VLP 20 µg” and “Commercial” were sampled for titration. Live virus was re-isolated in all sampled fish, both diseased and survivors, but with lower titers in the latter (Figure 8B).

NNV RNA1 was detected by RT-qPCR in all sampled dead and survivors in both Study 1 and 2, except for one survivor in Study 2 receiving the VLP high dose in Group 2 (challenged 1 m.p.v.) that was negative (Table 3). In Study 2, Group 1, the treatments “6 µg VLP”, “6 µg VLP + adj”, “commercial vaccine”, “PBS + adj”, and “PBS” did not have six survivors, hence a lower number of survivors was analyzed (4, 1, 2, 1, 1, respectively). There was no significant difference in the relative amount of RNA1 between the treatments when compared in ANOVA (*p* = 0.81) (only performed for Group 2, because of the low number of analyzed fish in Group 1). The relative quantification of RNA1 in the brains of survivors in Study 2 is available in the supplementary material (Figure S4).

### 3.7. Immunohistochemistry (Study 1)

**Figure 9:**
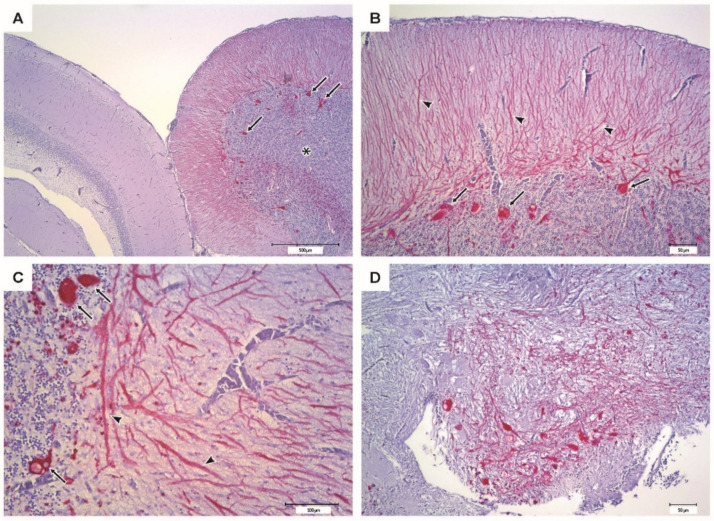
Immunohistochemistry of the encephalon from moribund sea bass (PBS, negative control group). Bright red coloring indicates the presence of RGNNV antigens. (**A**–**C**) Cerebellum (*) with intense immunostaining at different magnifications; the cortical of the optic lobes appears scarcely affected. Cerebellar Purkinje cell soma (arrows) appears strongly IHC positive, while their dendritic process (arrowheads) is also highlighted in the molecular layer. (**D**) Diencephalon showing intense immunostaining.

**Figure 10:**
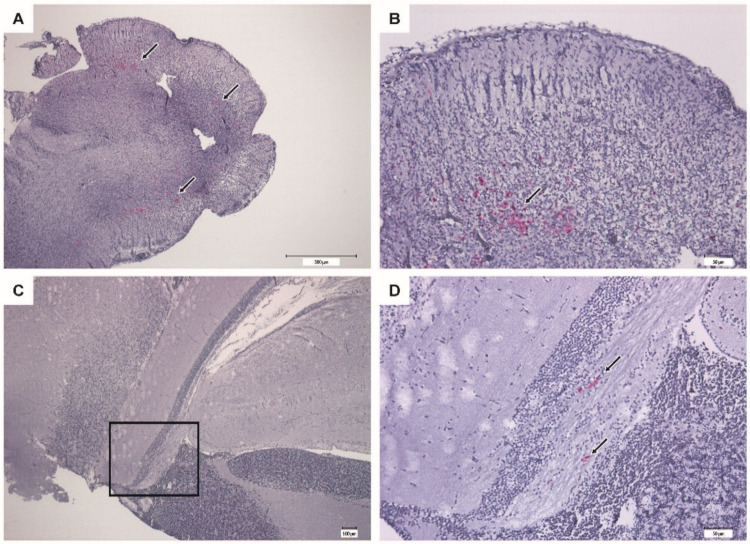
Immunohistochemistry of brain from vaccinated surviving sea bass (VLP 20 µg/fish). Bright red immunostaining indicates the presence of RGNNV antigens. (**A**,**B**) Cerebellum at different magnifications showing mild coloring (arrows). (**C**,**D**) Mesencephalon with scattered staining (arrows). (**D**) Higher magnification of the squared section in (**C**).

The IHC of brain from moribund fish revealed massive positive staining for RGNNV antigen in the cerebellum, diencephalon, and telencephalon (Figure 9). Sections from vaccinated survivors only revealed sporadic positive staining in the cerebellum, diencephalon and mesencephalon, where much fewer cells were stained (Figure 10).

### 3.8. Serology—Survivors (Study 1)

In Study 1, upon termination of the experiment, 10 survivors pr. group were blood sampled and tested in ELISA and SN. If there were fewer survivors, the number was reduced. The final number of samples and the results are presented in Table 4.

## 4. Discussion

VLPs have been shown to be effective vaccine candidates against various diseases in many veterinary species, including VNN in sea bass [26,28,29]. However, for VNN in sea bass, data on the duration of immunity and optimal formulation are lacking as well as a safe and up-scalable production method. In the present study, we have tested the efficacy and duration of immunity of a VLP vaccine based on betanodavirus capsid protein expressed in *P. pastoris*. The VLPs were successfully produced and purified. The characterization by Western blot and cryo-TEM confirmed correct size and morphology. We here report two independent vaccination trials in which different doses of the VLP vaccine were injected intraperitoneally into European sea bass with or without adjuvant followed by IM challenge with RGNNV at different times post-vaccination. 

Dose-dependent protection was observed in all experiments, with high survival among fish given 20 µg VLP and challenged 1 or 2 months later with RPS values of 67 and 73, respectively, and up to a 26 times greater chance of survival than the control group receiving PBS. Although not as significant, lower VLP vaccine doses also increased the chance of survival compared to PBS. These results support the previous findings of Thiéry et al. (2006) [29], who tested another betanodavirus VLP vaccine in sea bass and found a significant reduction in mortality at doses ≥ 1 µg VLP/fish (vaccinated IM) when the fish were challenged app. 1 month (700 dd) post-vaccination. In Trial 1, by Thiéry et al., which had comparable conditions to our studies, they obtained RPS = 71.1 when administering 20 µg VLP/fish, which is similar to the RPS values in both of our studies. However, the VLP vaccine prepared by Thiéry et al. was based on the expression of the NNV capsid protein in insect cells using a baculovirus vector and implied cumbersome purification steps. The baculovirus vector is a GMO, and may be hard to eliminate from the VLP preparation [30], hereby implicating some environmental safety issues related to registration for use in farmed fish. The Pichia-produced VLP preparations used in our study were cell free and obtained by a few up-scalable purification steps, making the approach suitable for future applied use [30]. To our knowledge, long-term protection following VLP- based vaccination of sea bass has not been examined earlier, and we here demonstrate that the protection persist until at least two months/1180 dd post-vaccination and to some degree also at 10 months/3600 dd post-vaccination (RPS 20 µg, 10 months = 13.6 (Table 3)), although the chance of surviving was significantly reduced at 10 months post-vaccination compared to 1 month post-vaccination (OR = 0.7 [0.7–0.8] (Table 2)). However, the 10 months long-term vaccination/challenge trial was compromised by several aspects. The very low survival of the negative control fish in Group 1 (PBS = 1.8% and PBS + adj = 1.7%) indicated that the dose of virus used for challenge had been too high and might have overwhelmed the protective immune mechanisms. This is supported by the fact that the commercial vaccine, which is registered to provide immunity for one year, performed poorly at this time point (4.0% survival). Furthermore, due to lack of options for higher water temperature in the used rearing facility, the fish were kept at 12 °C during the 10-month immunization period. As seen from the very limited growth of the fish, it may be assumed that this, for sea bass, rather low rearing temperature, might have delayed and/or reduced the immune response compared to fish kept at a physiologically more optimal temperature. Accordingly, Cecchini et al. (2002) found 24 °C to be the optimal water/body temperature for the development of antibody response in sea bass, with declining levels of antibodies at colder and warmer water temperatures [47]. For fish in general, it has been observed that low water temperatures delay or even inhibit the adaptive immune response [48]. As for the other vaccinated groups, we could detect specific antibodies by ELISA in plasma from the majority of the vaccinated fish kept at 12 °C at the time of challenge, but the quantitative effect of the low temperature on the immune response remains to be examined.

As stated above, specific antibodies were detected in a qualitative ELISA in all sampled fish receiving the VLP vaccine at all time-points, and at 2 months post-vaccination there were still measurable neutralizing antibodies. This corroborates with the VLPs being very immunogenic and stimulating a strong adaptive immune response [25]. Neutralizing antibodies were detected in all vaccinated fish (both VLP and commercial) in Study 1, with a dose-dependent response in the geometric mean titer (GMT) (Figure 6) peaking at Day 27 p.v. with a GMT of 320 in the 20 µg VLP decreasing with the lower doses. A slight decline was noted at Day 57 p.v, with a GMT of 289 in the 20 µg VLP dose and a similar decline in fish given lower doses. The apparent correlation between GMT and survival suggests a correlation with protection against disease at a neutralizing titer ≥ 279, and at least not lower than 200, as this was the mean titer in the medium dose group (5 µg/fish) which had significantly lower survival than the high-dose group. This is in agreement with a previous study in sevenband grouper (*Epinephelus septemfasciatus*) by Yamashita et al. (2009) [49]. In that study, fish were immunized with formalin inactivated RGNNV in different concentrations and significant protection correlated with mean serum neutralizing titer ≥197. However, this seemingly direct correlation between neutralizing titer and survival was not directly supported by the data on the individual fish in our study (Figure 8A). Here, fish with titers above 500 also developed clinical disease within the first 10 days p.c. The study only included 8–10 fish per group, and blood sampling 2 days before challenge might have compromised the ability of the fish to cope with the infection, but the overall lack of correlation between titer of neutralizing antibodies and protection was quite evident (Figure 8A). Thus, although there was a correlation between dose, GMT and protection, the relationship was not necessarily causal between GMT and protection. This suggests that other immune mechanisms are pivotal in the protection against disease development and warrants further studies. Valero et al. (2016) [15] detected an upregulation of genes related to cell-mediated cytotoxicity of CD8+ t-cells and interferon genes by RTqPCR in the gut of sea bass orally vaccinated against VNN with a DNA vaccine. ELISA and SN was negative, indicating a lack of circulating antibodies. Nonetheless, the orally vaccinated fish showed a protection when challenged IM with NNV, indicated by delay in onset of disease and a survival percentage of 45% compared to 0% in the non-vaccinated controls [15]. In another study with European Sea Bass vaccinated IM or IP with Atlantic Cod (AC)NNV VLPs, no specific IgM response was detected in ELISA although the fish were partially protected in an experimental IM challenge with RGNNV [21]. ACNNV is cross-reactive with RGNNV serotype, so antibodies raised against ACNNV VLP should bind to RGNNV.

A commercial oil adjuvanted vaccine with inactivated RGNNV as antigen was used as a positive reference control in our experiments. Although protection was seen in groups of fish receiving this vaccine, the effect was significantly lower than in the fish given 20 µg VLP. All RGNNV isolates belong to the same serotype [10], though genetic variability within the nucleocapsid gene among different RGNNV isolates has been reported [35,50] and the level of protection across this variability by inactivated/VLP vaccines has not been analyzed. Since information about the virus isolate used as antigen in the commercial vaccine is not disclosed by the supplier, and we only used one RGNNV isolate for challenge, it cannot be excluded that variability in, e.g., epitopes involved in triggering cellular immunity, may explain the observed differences in protection. Alternatively, the differences may be related to the amounts of antigen in the vaccines, since a clear dose–response relationship was observed in the VLP-vaccination trials reported here. Finally, the fish used in Study 2, Group 1, were only 5 g and the commercial vaccine is recommended for fish at 12 g and above, possibly to reduce the chance of adjuvant-related side effects.

Emulsification of aqueous vaccine antigens with oil adjuvants is often used to obtain optimal and prolonged immune response in fish vaccines for IP injection [51]. However, the mineral oil adjuvant used in this study did not influence the chance of survival. In fact, a lower rate of survival was seen in the adjuvanted groups, although it was not significantly different from the non-adjuvanted (*p* = 0.26). One of the benefits of using VLPs as vaccines is the relatively large immunogenic size, which corresponds to the size of the pathogen and apparently makes the adjuvant redundant. In a similar study, in which dragon groupers (*Epinephelus lanceolatus*) were vaccinated against RGNNV with a VLP vaccine, a positive effect of Freund’s complete adjuvant (FCA) was seen on the antibody response of vaccinated fish, measured by a capture ELISA 4 weeks after immunization [52]. However, the authors did not test whether this increase in antibodies correlated with higher survival, as the fish were not challenged. Other adjuvants, such as FCA, could be tested with the VLP to investigate if the dose can be lowered while still achieving the same good protection as with 20 µg/fish. However, the possible peritonitis and intra-abdominal lesions that can be induced by adjuvants administered IP can compromise both the welfare and growth of the fish [18,19,20]. Although sometimes necessary, it is therefore desirable not to use adjuvants if immune response and protection can be achieved without.

The lack of neutralizing antibodies in survivors, although positive for specific antibodies in ELISA, may reflect that the neutralizing antibodies, confirmed two days before the challenge, did actually neutralize the circulating virus and were not detectable at sampling time. However, challenge and infection would be expected to induce/boost a new production of antibodies, as would a booster vaccination. The SN is a biological assay comprising certain variability, and further experiments are needed to clarify the underlying mechanisms. To our knowledge, the course of neutralizing antibodies during and after the experimental infection of vaccinated sea bass has not been extensively investigated, although this is very relevant from a farm perspective, where it is likely that the fish is going to encounter the virus several times during a life span. Gye et al. (2018) [53] studied the kinetics of the antibody response in convalescent sevenband grouper upon reinfection with NNV but failed to detect neutralizing serum activity in survivors, supporting that other immune mechanisms are involved in protection.

Vaccinated fish surviving the infection still harbored live virus in the brain 28 days post-challenge, although at a much lower level than the moribund fish (Figure 8B). This was confirmed by immunohistochemistry staining for RGNNV, where only a few infected neurons were in survivors (Figure 10). Since the challenge was done by injection, these results do not necessarily reflect natural conditions, and experimental challenge by immersion or cohabitation will be needed to determine whether the VLP-based vaccine can protect the fish not only against disease but also against infection.

## 5. Conclusions

In the current study, we present an RGNNV VLP able to induce a dose-dependent response of neutralizing antibodies in European sea bass. All tested doses of VLP offered protection against experimental IM challenge with 10^5^ TCID_50_/fish RGNNV, with the high-dose VLP (20 µg/fish) inducing the best protection. Furthermore, by modelling the probability of survival 1 and 10 months after vaccination, we find that both doses of VLP improve the chance of surviving significantly, compared to the group receiving PBS, although the overall chance of surviving is reduced at 10 months post-vaccination. The adjuvant did not improve the probability of surviving. Notably, survivors tested positive for virus in the brain; therefore, the VLP vaccine provides protection from clinical signs and mortality but not from infection. Even though it seems that there is a correlation between the level of RGNNV-neutralizing antibodies and survival, survival may not solely be due to neutralizing antibodies; rather, some other immune mechanisms may play a role as well.

## Figures and Tables

**Table 1:** Overview of details and analysis in Study 1 and 2.

	Study 1	Study 2	
		Group 1	Group 2
Vaccination			
Fish size	29 g (±2 g)	5 g (±2 g)	14 g (±2 g)
Treatments	VLP 1.25, 5 and 20 µg/fish Commercial vaccine PBS	VLP 6 (±adj) and 20 µg/fish Commercial vaccine PBS (±adj)	
Serology	ELISA Serum neutralization	ELISA	
Immunization period	2 months at 20 °C/ 1180 dd ^1^	10 months at 12 °C/ 3600 dd	1 month at 19 °C/ 570 dd
Challenge			
RGNNV dose	2 × 10^5^ TCID_50_/fish	10^5^ TCID_50_/fish	
Moribund (detection of NNV)	Isolation and titration of live RGNNV from brain tissue on E-11 cells RT-qPCR Immunohistochemistry	RT-qPCR	
Survivors (detection of NNV)	Isolation and titration of live RGNNV from brain tissue on E-11 cells RT-qPCR Immunohistochemistry	RT-qPCR (with relative quantification (2^-∆^^∆^^cq^-method))	
Statistical analysis	Pearson’s Chi-squared	Logistic regression	

^1^ dd = degree days.

**Table 2:** Odds ratio (OR) of survival in Study 2.

Factor	OR [95% CI]
PBS	1
Commercial vaccine	3.5 [1.7–7.2]
VLP 6 µg	4.8 [2.6–9.1]
VLP 20 µg	12.1 [5.9–26.2]
Time = 10 months	0.7 [0.7–0.8]

Note: OR calculated as exp(β) from the glm model.

**Table 3:** Survival data and re-isolation/detection of virus.

	Treatment	Survival (%) Replicate Tanks	Survival (%) Combined [CI] ^1^	RPS ^2^	Moribund Fish		Survivors	
					Cells	RT-qPCR	Cells	RT-qPCR
Study 1								
Time = 2 months	PBS		0.0 ^a^		5/5	5/5	NT	NT
	Commercial		18.8 ^b^ [5.2–32.3]	18.8	4/4	4/4	3/3	3/3
	VLP 1.25 µg		35.0 ^b^ [20.2–49.8]	35.0	3/3	3/3	NT	NT
	VLP 5 µg		8.6 ^b^ [0–17.8]	8.6	5/5	5/5	NT	NT
	VLP 20 µg		73.5 ^c^ [58.7–88.4]	73.5	4/4	4/4	4/4	4/4
	Control ^3^		100		NT	NT	NT	NT
Study 2								
Time = 10 months	PBS	0.0; 3.6	1.8 * [0.0–5.2]		NT	6/6	NT	1/1
	PBS + adj	3.1; 0.0	1.7 * [0.0–5.0]		NT	6/6	NT	1/1
	Commercial	4.0; 4.0	4.0 ** [0.0–9.4]	2.3	NT	6/6	NT	2/2
	VLP 6 µg	11.1; 3.7	7.4 ** [0.4–14.4]	5.8	NT	6/6	NT	4/4
	VLP 6 µg + adj	3.2; 0.0	1.7 ** [0.0–4.9]	−0.1	NT	6/6	NT	1/1
	VLP 20 µg	8.0; 21.4	15.1 ** [5.5–24.7]	13.6	NT	6/6	NT	6/6
Time = 1 month	PBS	21.7; 29.2	25.5 ^#,a^ [13.2–38.0]		NT	6/6	NT	6/6
	PBS + adj	17.4; 20.0	18.8 ^#^ [7.7–29.8]		NT	6/6	NT	6/6
	Commercial	55.0; 48.0	51.1 ^##,b^ [36.5–65.7]	34.3	NT	6/6	NT	6/6
	VLP 6 µg	64.3; 60.0	62.1 ^##,b^ [44.4–79.7]	49.1	NT	6/6	NT	6/6
	VLP 6 µg + adj	50.0; 70.0	59.5 ^##,b,c^ [43.6–75.3]	45.6	NT	6/6	NT	6/6
	VLP 20 µg	73.7; 77.3	75.6 ^##,c,d^ [62.5–88.8]	67.2	NT	6/6	NT	5/6
	Control		95.2 [91.5–99.0]		NT	0/6	NT	NT

Survival percent (moribund/(total − censored) * 100%) at the end of the experiments (Day 28 (Study 1) or Day 27 (Study 2)) and the number of fish where the virus was re-isolated from the brain by inoculation on E11 cells (cells) or by RTqPCR (RT-qPCR) as described in ”methods” (positive/tested). NT = Not tested. ^1^ 95% confidence interval: $p\left(surv\right)\mp 1.96\times \sqrt{\frac{p\left(surv\right)\times \left(1-p\left(surv\right)\right)}{n}}$; ^2^ RPS = (1 − (mort%(treatment)/mort%(PBS)) * 100 [46]; ^3^ non-challenged controls; ^a,b,c,d^ different letters indicate significantly different survival at each timepoint (Pearson’s chi square test); */**/^#^/^##^ different symbols indicate significantly different odds of surviving (logistic regression).

**Table 4:** Serum samples from survivors and the results from ELISA and Serum neutralization (SN).

Treatment	*n*	ELISA Pos	SN > 20
VLP 20 µg	10	10/10	0/10
VLP 5 µg	3	3/3	0/3
VLP 1.25 µg	10	10/10	0/10
Commercial	6	6/6	0/6
PBS	0	0	0

## Data Availability

The data presented in Study 1 are available on request from the corresponding author. The data are not publicly available due to other investigations ongoing. The data presented in Study 2 are openly available in DTU Data at doi:10.11583/DTU.13947395.

## Supplementary Materials

The following are available online at https://www.mdpi.com/article/10.3390/vaccines9050447/s1, Figure S1: Sea Bass from Study 1 tagged with fluorescent elastomers (VIE tag—Northwest Marine Technology) in UV light, Figure S2: Gel and Western blot, Figure S3: Predicted p(surv)., Figure S4: Relative amount of RGNNV RNA1.
