# Supplementary material for: Long-Term Protection and Serologic Response of European Sea Bass Vaccinated with a Betanodavirus Virus-Like Particle Produced in Pichia pastoris
Source: Vaccines (Basel). 2021 May 2;9(5):447. doi: 10.3390/vaccines9050447 (PMC8147411; doi:10.3390/vaccines9050447)
Supplement: Supplementary file 1 [file vaccines-09-00447-s001.zip › vaccines-1184138-supplementary.pdf]

Supplementary material to Barsøe et al. (2021): "Long-term protection and serologic response of European sea bass vaccinated with a betanodavirus Virus-like Particle produced in *Pichia pastoris*"

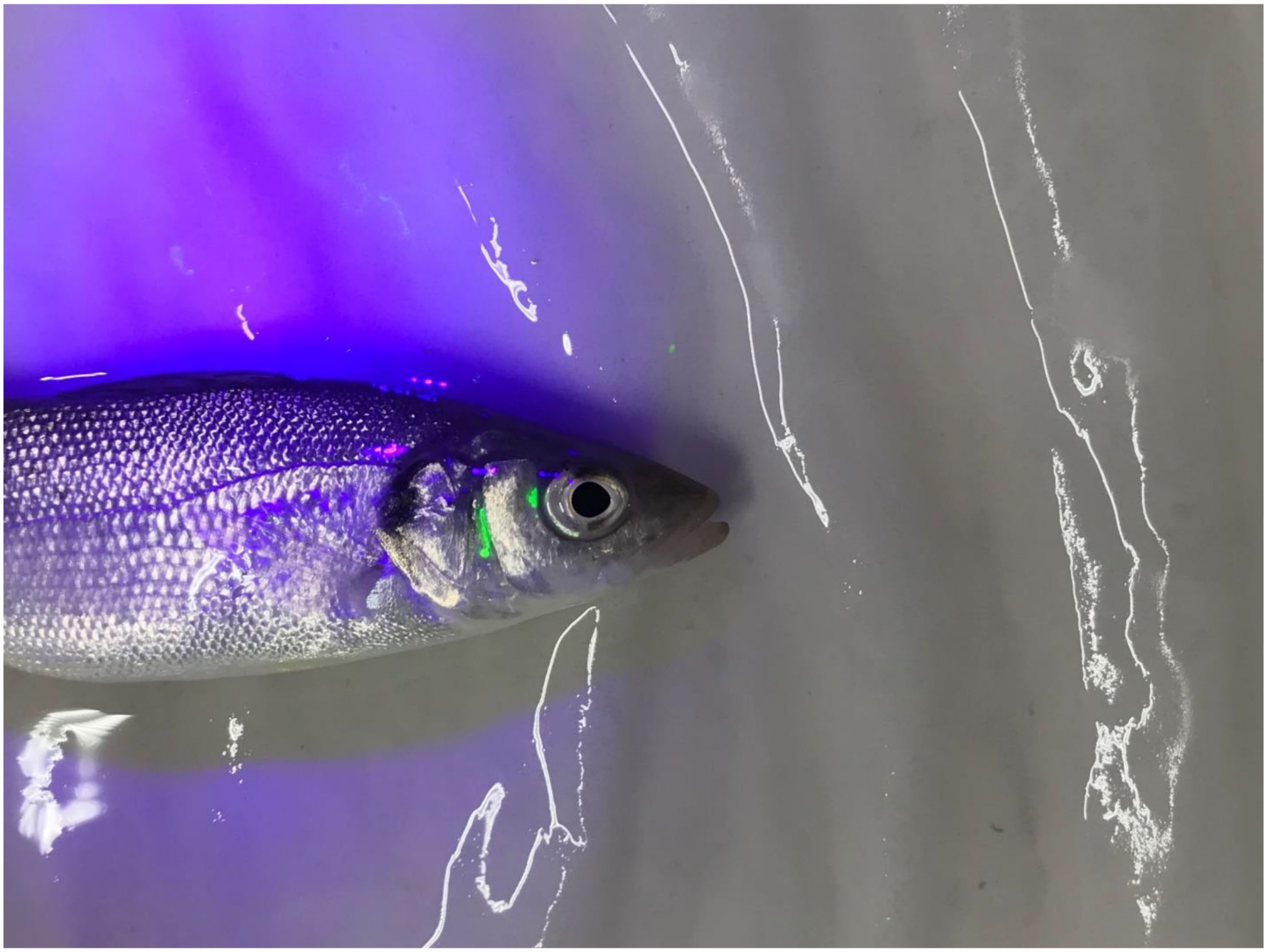

Figure S1. Sea Bass from Study 1 tagged with fluorescent elastomers (VIE tag - Northwest Marine Technology) in UV light.

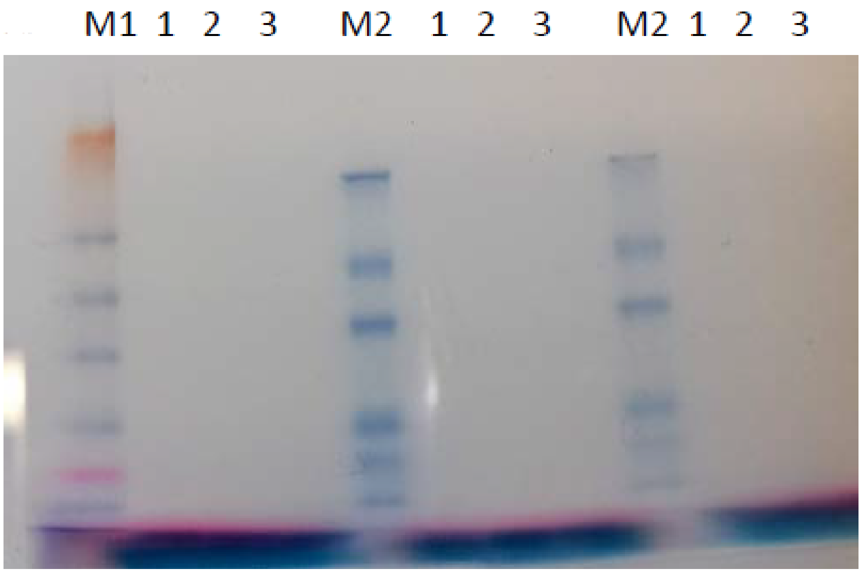

(**a**)

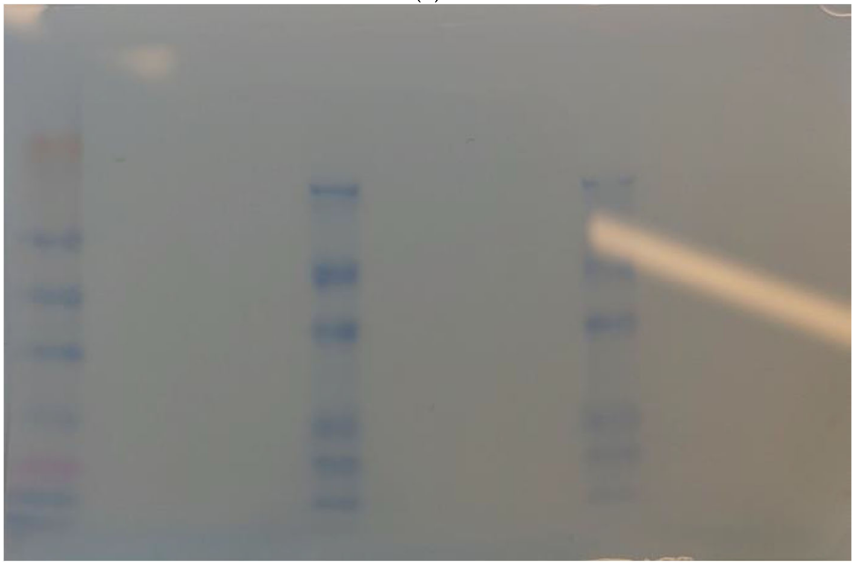

(**b**)

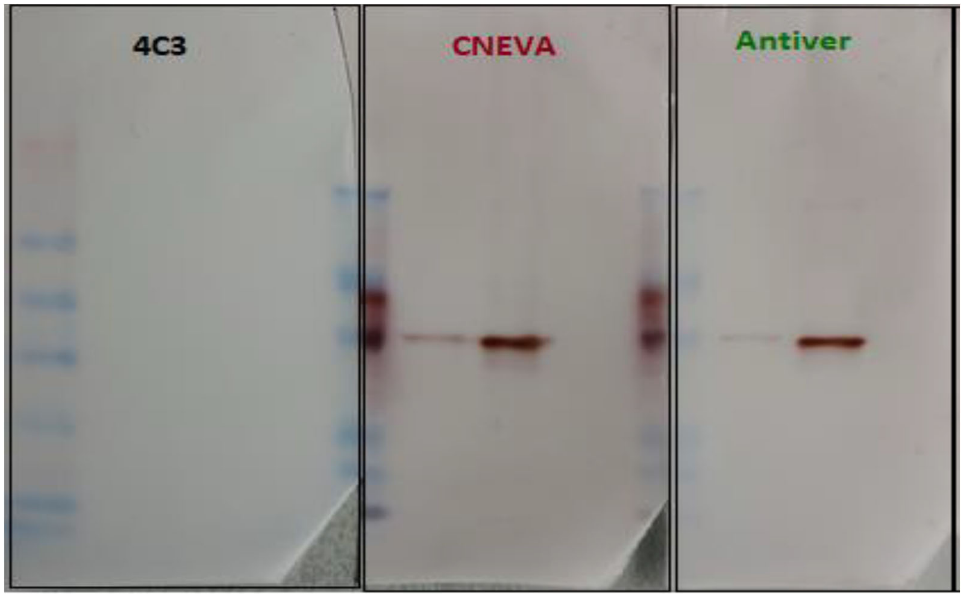

(**c**)

Figure S2. Gel and Western blot (A) Gel before transfer. (**B)** Membrane after transfer. (**C**) Membrane after staining. Note, that the membrane was cut in three and stained with three different primary antibodies antibodies ("4C3","CNEVA" and "Antiver"), where only the part stained with "Antiver" containing marker "M2" was included in the paper. Lane: (1) RGNNV (strain 283.2009), (2) RGNNV VLP, (3) MilliQ H2O. Marker M1 (BIO-RAD, precision protein standards, 113–21.5 kDa, low range, 6 bands, cat.nr 161-0305, picture below1:)

M1 (BIO-RAD, precision protein standards, 113–21.5 kDa, Broad Range, 6 bands):

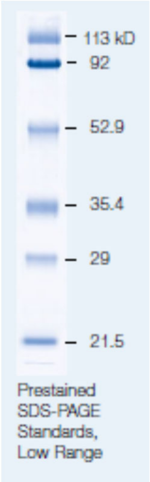

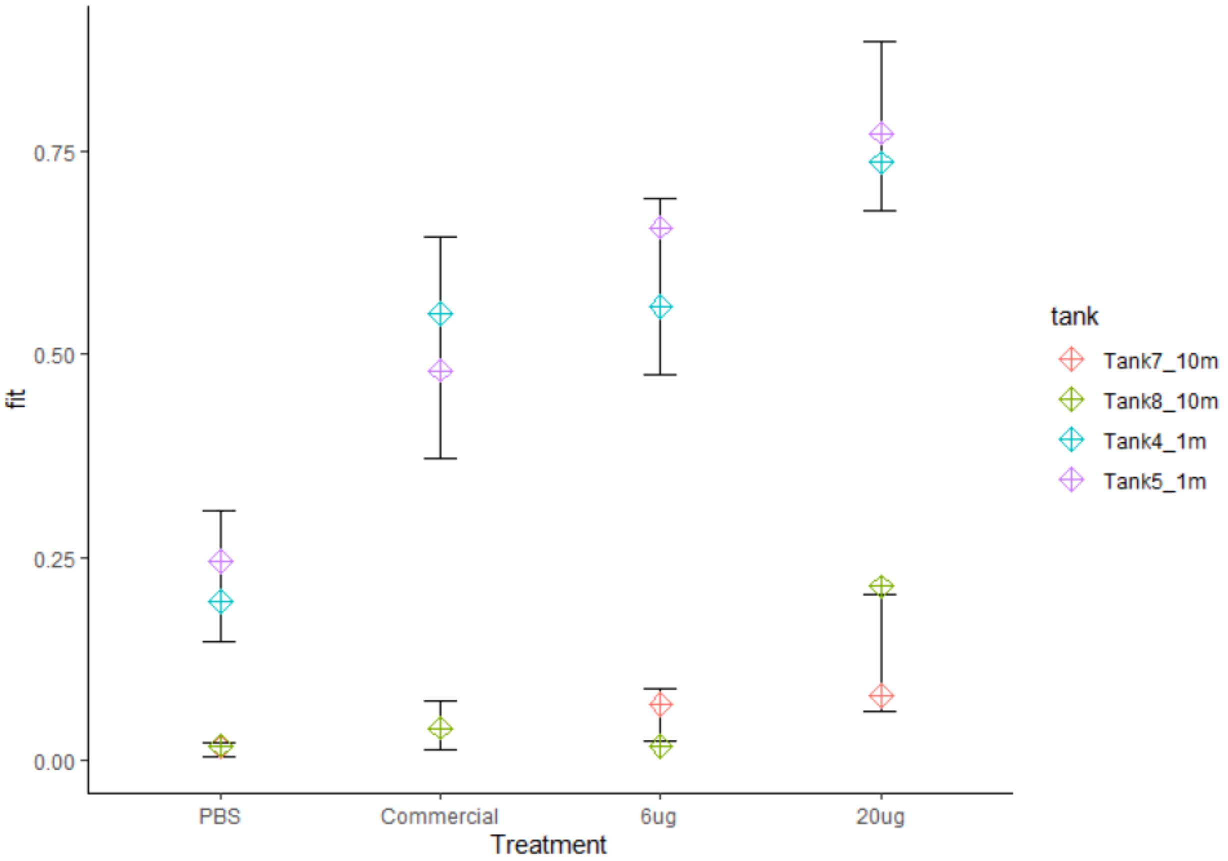

Figure S3. Predicted p(surv). 95% confidence interval for the treatments PBS, 6ug, 20ug and the commercial vaccine at Time = 10 months (10m) (lower interval and Time= 1 month (1m) (higher interval). The colored squares are the observed p(surv) for the two replicate tanks.

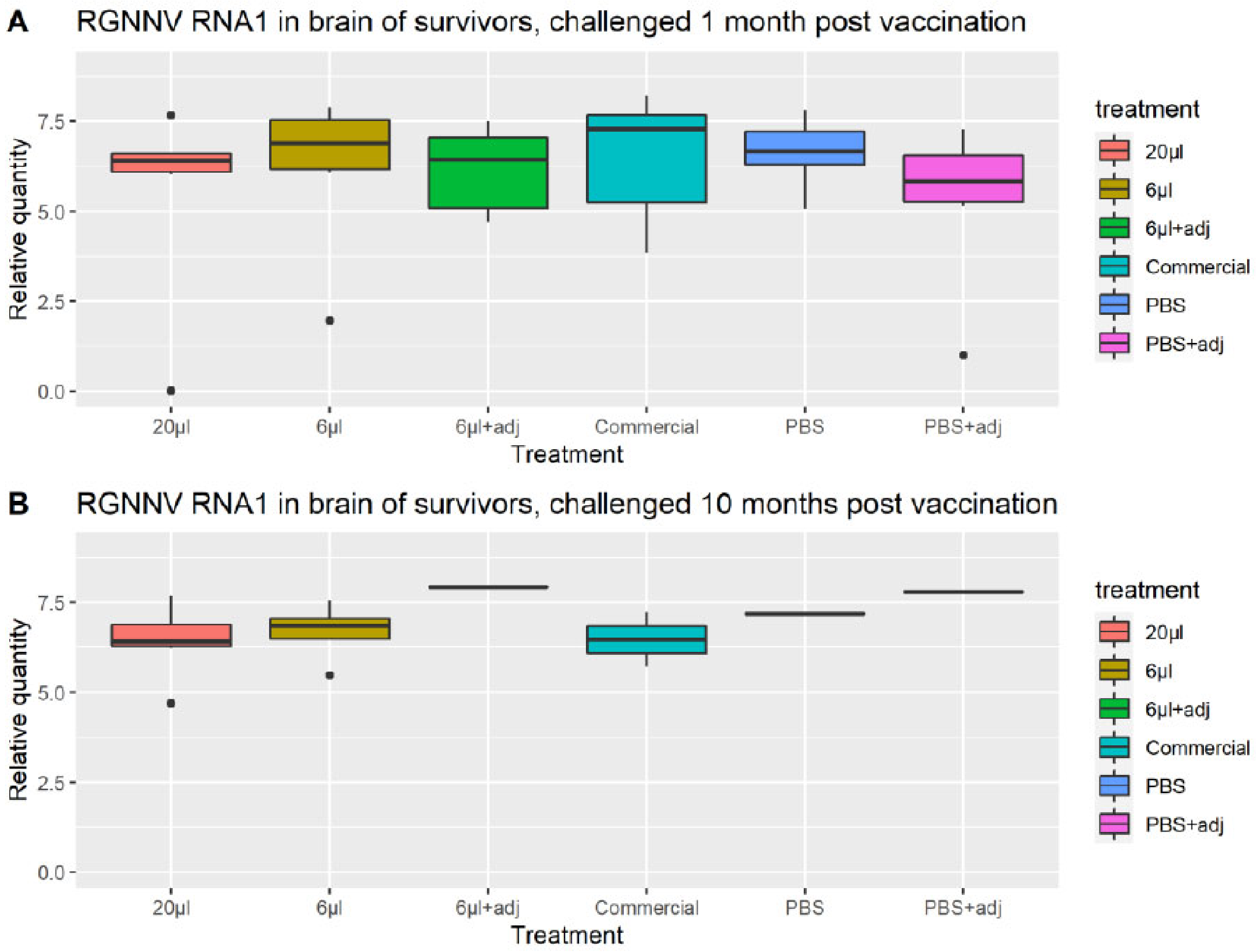

Figure S4. Relative amount of RGNNV RNA1. Brain tissue of vaccinated survivors (Study 2) challenged either 1 (A) or 10 (B) months post vaccination was analyzed by RTqPCR. The relative amount was determined by the $2^{-\Delta\Delta Cq}$-method as previously described [32]; $\Delta\Delta Cq = \Delta Cq_{sample} - \Delta Cq_{calibrator}$, where $\Delta Cq = Cq_{RNA1} - Cq_{Ef1\alpha}$. The sample with least amount of virus RNA (highest Cq value) was used as calibrator (marked with a red circle). Numbers per group as specified in the text (1-6, depending on number of survivors).
